# Early predicting 30-day mortality in sepsis in MIMIC-III by an artificial neural networks model

**DOI:** 10.1186/s40001-022-00925-3

**Published:** 2022-12-17

**Authors:** Yingjie Su, Cuirong Guo, Shifang Zhou, Changluo Li, Ning Ding

**Affiliations:** Department of Emergency Medicine, The Affiliated Changsha Central Hospital, Hengyang Medical School, University of South China, NO. 161 Shaoshan South Road, Changsha, 410004 Hunan China

**Keywords:** Artificial neural networks, Sepsis, Mortality, MIMIC-III

## Abstract

**Objective:**

Early identifying sepsis patients who had higher risk of poor prognosis was extremely important. The aim of this study was to develop an artificial neural networks (ANN) model for early predicting clinical outcomes in sepsis.

**Methods:**

This study was a retrospective design. Sepsis patients from the Medical Information Mart for Intensive Care-III (MIMIC-III) database were enrolled. A predictive model for predicting 30-day morality in sepsis was performed based on the ANN approach.

**Results:**

A total of 2874 patients with sepsis were included and 30-day mortality was 29.8%. The study population was categorized into the training set (*n* = 1698) and validation set (*n* = 1176) based on the ratio of 6:4. 11 variables which showed significant differences between survivor group and nonsurvivor group in training set were selected for constructing the ANN model. In training set, the predictive performance based on the area under the receiver-operating characteristic curve (AUC) were 0.873 for ANN model, 0.720 for logistic regression, 0.629 for APACHEII score and 0.619 for SOFA score. In validation set, the AUCs of ANN, logistic regression, APAHCEII score, and SOFA score were 0.811, 0.752, 0.607, and 0.628, respectively.

**Conclusion:**

An ANN model for predicting 30-day mortality in sepsis was performed. Our predictive model can be beneficial for early detection of patients with higher risk of poor prognosis.

## Introduction

Sepsis, as a syndrome of organ dysfunction induced by a dysregulated response to infection, was one of major causes leading to high mortality and poor clinical outcomes in intensive care unit(ICU) [1, 2]. Studies reported that the short-term and long-term mortality of sepsis varied from 20 to 50% [3–5]. Hence, early identifying sepsis patients who had higher risk of poor prognosis was extremely important for physicians so they can do some intervention and timely managements to improve the clinical outcomes [6].

Artificial neural networks (ANN), as a type of machine learning algorithm, have been applied widely for medical researches [7–9]. One study with a total of 21,892 cases showed that ANN model had a good performance for predicting 14-day hospital readmission with pneumonia [10]. Another recent research on cancer demonstrated that ANN model was capable of simultaneously predicting the multiple co-occurring symptoms including the risk of pain, psychological disorders and lack of well-being [11]. In the COVID-19 pandemic, scientific researchers in Brazil applied the ANN model to easily make daily and cumulative forecasts for cases and deaths so that government officials and medical agencies could do actions more agilely and reliably [12].

In the present study, we aimed to explore the capability of ANN model in predicting clinical outcomes in sepsis based on the publicly accessible database of Medical Information Mart for Intensive Cart III (MIMIC-III).

## Methods

### Database and patients

MIMIC-III database is a US-based critical care public database. Clinical and laboratory data associated with 53,423 age ≥ 16 patients from 2001 to 2012 and 7870 neonates from 2001 to 2008 admitted in ICU were documented [13]. The database mainly included charted events such as demographics, vital signs, laboratory tests, vital status, medications, image reports, and clinical outcomes.

All patients with sepsis (ICD9 code: 99,591) in MIMIC-III (version 1.4) were enrolled in this study. Exclusion criteria included as follows: patients with missing > 5% individual data and age less than 18.

### Data extraction

From the MIMIC-III database, the following general variables were extracted for the first 24 h after ICU admission: age at the time of hospital admission, gender, admission type, marital status, ethnicity, ICU department, comorbidities (renal disease, coronary artery disease (CAD), diabetes, and hypertension), sequential organ failure assessment (SOFA) score and acute physiology and chronic health evaluation (APACHEII) score. The length of stay (LOS) in ICU and in-hospital mortality were also collected.

Clinical and laboratory variables which were recorded within 24 h after admission were also extracted including systolic blood pressure (SBP), diastolic blood pressure (DBP), heart rate (HR), respiratory rate (RR), white blood cells (WBC), neutrophils, lymphocytes, sodium, chloride, platelet (PLT), red cell volume distribution width (RDW), mean corpusular volume (MCV), hematocrit, glucose, prothrombin time (PT), partial thrombin time (PTT), albumin, alanine aminotransferase (ALT), aspartate aminotransferase (AST), total bilirubin, urea nitrogen, creatinine, lactate, total calcium, and anion gap. NLR is defined as the ratio of neutrophils to lymphocytes. Multiple multivariable imputations were utilized for addressing missing data to maximize statistical power and minimize bias.

### Statistical analysis

Descriptive statistics included as follow: proportions and frequencies were used for categorical variables, while medians, mean (SD), and interquartile ranges (IQRs) were used for continuous variables. Chi-squared test or Mann–Whitney U test were utilized for the comparison between the survivor group and the nonsurvivor group.

First, univariable analysis was applied for identifying variables which were significantly different between the two groups. Then, those variables were enrolled to construct the predictive model by multivariable logistic regression. At last, the receiver-operator characteristic (ROC) analysis for predicting 30-day mortality was performed and the area under the curve (AUC) estimates were calculated. The analyses of accuracy, sensitivity, and specificity were also done for evaluating the predictive performance of different models. The best threshold values of variables were confirmed by the Youden Index (sensitivity+specificity-1). The value of each variable with the maximum Youden Index was the best threshold value.

Statistical analysis was performed by using SPSS software (version 26). A *p* value of < 0.05 was considered as statistically significant.

### ANN model

For our ANN model, a multilayer perception with back propagation algorithm was the applied architecture [14, 15]. The basic structure of ANN had three layers including the input layer, the hidden layer and the output layer (Fig. 2). The variables which showed significant differences between the survivor group and nonsurvivor group by using univariate analysis were enrolled in the input layer. In Fig. 2, our ANN was composed with 1 input layer consisting of 12 nodes, 1 hidden layer consisting of 6 nodes, and 1 output layer consisting of 2 nodes.

The study population was categorized into the training set (*n* = 1689) and the validation set (*n* = 1176) was based on the ratio of 6:4 by simple randomization using R software function of set.seed (), respectively. We applied an oversampling algorithm method to deal with the imbalance between training set and validation set [16]. The training set was utilized to construct models and the validation set was used to test the predictive performance of the models (Table 2). The predictive performance of ANN was analyzed by averaging the 30-day mortality from the fivefold cross-validation [11]. In addition, the average accuracy, sensitivity, and specificity were calculated. The predictive performances of ANN, logistic regression, APACHEII, and SOFA scores were compared for training set and validation set were compared. ANN model was performed with PyTorch (version1.2.0).

## Results

### General characteristics of sepsis in MIMIC-III

**Fig. 1:**
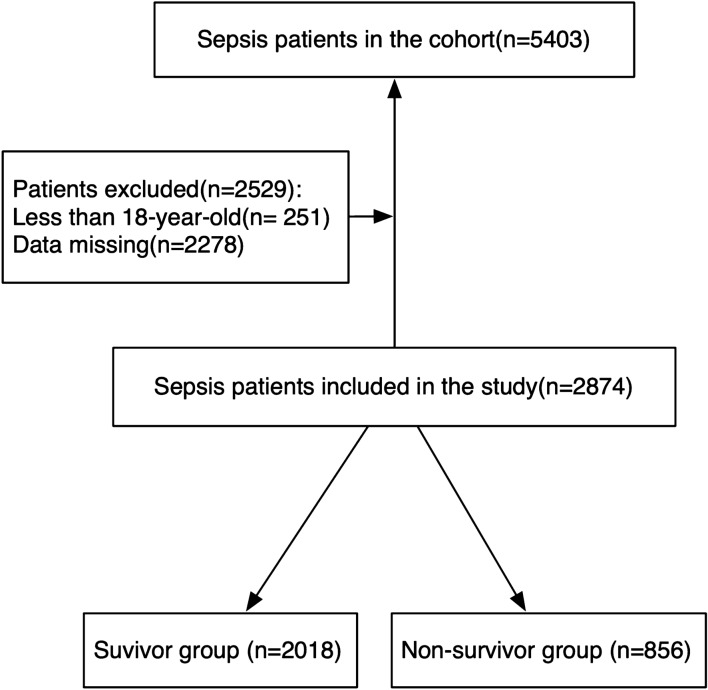
Flow chart for patients enrollment and study design

At first, a total of 5403 patients with sepsis were enrolled. Based on the exclusion criteria, 2874 patients were included in our study (Fig. 1). The 30-day mortality was 29.8%. The median age of the cohort was 67, and males accounted for 55.7% in total. Among marital status, the proportions of divorced, married, single and widow individuals were 6.8%, 44.5%, 28.4%, and 15.4%, respectively. Most of the patients were white (72.7%). 96% of patients were admitted in emergency and more than a half were transferred in MICU (65.9%). Among comorbidities, the proportions of renal disease, CAD, diabetes and hypertension were 8.4%, 15.9%, 5.4%, and 37.9%, respectively. The median scores of SOFA and APACHE in the cohort were 2 and 14, respectively. The median days of LOS in ICU and hospital were 3 and 8, respectively (Table 1).

**Table 1:** General characteristics of sepsis in MIMIC-III

Variables	
Number of patients(*n*)	2874
Age(years)	67 (56–80)
Gender (*n*, %)	
Male	1602 (55.7%)
Female	1272 (44.3%)
Marital status (*n*, %)	
Divorced	195 (6.8%)
Married	1279 (44.5%)
Single	816 (28.4%)
Widow	442 (15.4%)
Others	142 ((4.9%)
Ethnicity (*n*, %)	
Asian	77 (2.7%)
White	2089 (72.7%)
Black/American	273 (9.5%)
Hispanic/Latino	100 (3.5%)
Others	335 (11.6%)
Department (*n*, %)	
CCU	238 (8.3%)
MICU	1894 (65.9%)
SICU	428 (14.9%)
TICU	222 (7.7%)
CSRU	92 (3.2%)
Admission type (*n*, %)	
Elective	77 (2.7%)
Urgent	38 (1.3%)
Emergency	2759 (96.0%)
Comorbidities (*n*, %)	
Renal disease	241 (8.4%)
CAD	457 (15.9%)
Diabetes	155 (5.4%)
Hypertension	1089 (37.9%)
Scoring system	
APACHEII	14 (11–17)
SOFA	2 (1–4)
Clinical outcomes	
LOS in ICU (days)	3 (1–8)
LOS in hospital(days)	8 (5–17)
Mortality (*n*, %)	
30-day mortality	856 (29.8%)

*SOFA* sequential organ failure assessment, *APACHE* acute physiology and chronic health evaluation, *CAD* coronary artery disease; *LOS* length of stay, *ICU* intensive care unit

### Baseline characteristics of training and validation tests

Table 2 demonstrated the general characteristics of training and validation. Except for diabetes (*P* = 0.021), there was no significant difference in other variables including age (*P* = 0.213), gender (*P* = 0.994), DBP (*P* = 0.310), SBP (*P* = 0.763), HR (*P* = 0.122), RR (*P* = 0.148), renal disease (*P* = 0.930), CAD(*P* = 0.542), hypertension (*P* = 0.774), PLT (*P* = 0.849), AST (*P* = 0.303), sodium(*P* = 0.931), glucose (*P* = 0.194), chloride (*P* = 0.510), MCV (*P* = 0.096), ALT(*P* = 0.420), neutrophils (*P* = 0.144), urea nitrogen(*P* = 0.617), PTT(*P* = 0.886), hematocrit (*P* = 0.355), PT (*P* = 0.949), anion gap (*P* = 0.070), RDW (*P* = 0.612), lymphocytes (*P* = 0.063), WBC (*P* = 0.089), NLR (*P* = 0.088), total calcium (*P* = 0.381), lactate (*P* = 0.790), albumin (*P* = 0.169), creatinine (*P* = 0.893), total bilirubin (*P* = 0.743), APACHEII (*P* = 0.581), SOFA (*P* = 0.671), LOS in hospital (*P* = 0.386) and 30-day mortality (*P* = 0.153).

**Table 2:** Baseline characteristics of training and validation sets

Variables	Training set (*n* = 1698)	Validation set (*n* = 1176)	*P* value
Age (IQR, year)	66 (56–80)	67 (56–79)	0.213
Gender (*n*, %)			0.994
Male	939 (55.3%)	663 (56.3%)	
Female	759 (44.7%)	513 (43.7%)	
Vital signs			
DBP (mmHg)	63 (51–73)	63 (51–72)	0.310
SBP (mmHg)	114 (98–131)	115 (97–131)	0.763
HR (beats/min)	97 (81–111)	97 (83–113)	0.122
RR (beats/min)	21 (16–24)	20 (16–24)	0.148
Comorbidities			
Renal disease (*n*, %)	149 (8.8%)	92 (7.8%)	0.930
CAD (*n*, %)	260 (15.3%)	197 (16.7%)	0.542
Diabetes (*n*, %)	103 (6.1%)	52 (4.4%)	0.021
Hypertension (*n*, %)	636 (37.5%)	453 (38.5%)	0.774
Laboratory characteristics			
PLT (*10^9^/L)	237.0 (137.0–310.0)	245.0 (148.0–320.0)	0.894
AST (IU/L)	193.0 (24.0–83.0)	174.0 (22.0–73.0)	0.303
Sodium (mmol/L)	137.0 (134.0–141.0)	137.0 (134.0–141.0)	0.931
Glucose (mg/dL)	151.0 (101.7–163.0)	155.0 (104.7–164.2)	0.194
Chloride (mmol/L)	102.0 (98.0–107.0)	101.0 (97.0–106.0)	0.510
MCV (fL)	91.0 (86.0–96.0)	90.0 (86.0–95.0)	0.096
ALT (IU/L)	107.0 (17.0–59.0)	98.0 (16.0–57.0)	0.420
Neutrophils (%)	78.0 (74.1–89.1)	77.0 (73.0–89.0)	0.144
Urea Nitrogen (mg/dL)	36.0(17.0–46.0)	35.0 (18.0–46.0)	0.617
PTT (s)	35.0 (27.2–38.0)	34.0 (26.9–37.1)	0.886
Hematocrit (%)	33.0 (28.8–37.5)	34.0 (29.6–38.1)	0.355
PT(s)	18.0 (13.3–19.1)	18.0 (13.2–18.4)	0.949
Anion Gap (mmol/L)	16.0 (14.0–19.0)	16.0 (13.0–19.0)	0.070
RDW (%)	15.0 (14.1–17.1)	15.0 (14.1–16.9)	0.612
Lymphocytes (%)	11.0 (4.3–13.3)	11.0 (4.2–14.0)	0.063
WBC (*10^9^/L)	13.0 (7.7–17.5)	12.0 (7.5–16.5)	0.089
NLR	15.0 (5.5–18.9)	15.0 (5.4–19.2)	0.088
Total calcium (mg/dL)	8.0 (7.5–8.8)	8.0 (7.4–8.8)	0.381
Lactate (mmol/L)	2.0 (1.4–3.2)	2.0 (1.4–3.4)	0.790
Albumin (g/dL)	2.0 (2.5–3.4)	2.0 (2.4–3.4)	0.169
Creatinine (mg/dL)	1.0 (0.9–2.2)	1.0 (0.9–2.4)	0.893
Total bilirubin (mg/dL)	2.0 (0.4–1.5)	1.0 (0.4–1.5)	0.743
Scoring system			
APACHEII (IQR)	13 (11–17)	14 (11–17)	0.581
SOFA (IQR)	2(1–4)	2 (1–4)	0.671
Clinical outcomes			
LOS in hospital (days)	13 (5–17)	13 (5–18)	0.386
30-day mortality (*n*, %)	526 (30.9%)	330 (28.1%)	0.153

*SBP* systolic blood pressure, *DBP* diastolic blood pressure, *HR* heart rate, *RR* respiratory rate, *CAD* coronary artery disease, *WBC* white blood cells, *PLT* platelet, *RDW* red cell volume distribution width, *PT* prothrombin time, *PTT* partial thrombin time, *ALT* alanine aminotransferase, *AST* aspartate aminotransferase, *SOFA* sequential organ failure assessment, *APACHE* acute physiology and chronic health evaluation, *LOS* length of stay, *IQR* interquartile ranges, *MCV* mean corpusular volume, *NLR* is defined as the ratio of neutrophils to lymphocytes

### Multivariable logistic regression analysis

In Table 3, significant differences were showed in variables including age (*P* < 0.001), AST (*P* < 0.001), MCV (*P* = 0.001), ALT (*P* < 0.001), urea nitrogen (*P* < 0.001), PTT (*P* < 0.001), PT (*P* < 0.001), RDW (*P* < 0.001), lactate (P < 0.001), albumin (*P* < 0.001) and total bilirubin (*P* < 0.001) between two groups in the training set.

**Table 3:** Comparison of variables between survivor and nonsurvivor groups in training set

Variables	Survivor (*n* = 1172)	Non-survivor (*n* = 526)	*P* value
Age (IQR, year)	65 (54–77)	70 (60–83)	< 0.001
Gender			0.539
Male (*n*, %)	645 (55.8%)	294 (55.9%)	
Female (*n*, %)	527 (44.2%)	232 (44.1%)	
Vital signs			
DBP (mmHg)	63 (52–73)	62 (49–72)	0.348
SBP (mmHg)	115 (98–130)	113 (97–131)	0.185
HR (beats/min)	96 (81–110)	98 (82–112)	0.187
RR (beats/min)	20 (16–24)	21 (17–25)	0.223
Comorbidities			
Renal diseases (*n*, %)	96 (8.02%)	53 (10.64%)	0.117
CAD (*n*, %)	172 (14.68%)	88 (16.73%)	0.287
Diabetes (*n*, %)	74 (6.31%)	29 (5.51%)	0.728
Hypertension (*n*, %)	441 (37.62%)	195 (37.07%)	0.637
laboratory characteristics			
PLT (*10^9^/L)	234.0 (151.0–313.0)	230.0 (122.7–302.0)	0.121
AST (IU/L)	109.0 (23.0–70.0)	374.0 (27.0–128.5)	< 0.001
Sodium (mmol/L)	137.0 (134.0–140.0)	137.0 (133.0–141.0)	0.757
Glucose (mg/dL)	154.0 (103.0–163.0)	146.0 (99.0–165.0)	0.164
Chloride (mmol/L)	102.0 (98.0–107.0)	102.0 (97.0–107.0)	0.751
MCV (fL)	90.0 (86.0–95.0)	92.0 (88.0–98.0)	0.001
ALT (IU/L)	73.0 (16.0–55.0)	180.0 (17.0–74.5)	< 0.001
Neutrophils (%)	79.0 (74.4–89.0)	77.0 (73.7–89.5)	0.125
Urea Nitrogen (mg/dL)	32.0 (16.0–40.0)	44.0 (22.0–57.0)	< 0.001
PTT (s)	34.0 (26.7–35.7)	38.0 (28.4–41.5)	< 0.001
Hematocrit (%)	33.0 (29.0–37.6)	33.0 (28.4–37.0)	0.248
PT (s)	17.0 (13.2–17.5)	20.0 (13.9–22.0)	< 0.001
Anion Gap (mmol/L)	16.0 (13.0–19.0)	16.0 (14.0–20.0)	0.267
RDW (%)	15.0 (13.9–16.6)	16.0 (14.7–18.3)	< 0.001
Lymphocytes (%)	10.0 (4.4–13.3)	11.0 (4.0–13.3)	0.499
WBC (*10^9^/L)	13.0 (7.8–17.0)	13.0 (7.3–18.6)	0.083
NLR	15.0 (5.6–18.8)	15.0 (5.2–18.8)	0.875
Total calcium (mg/dL)	8.0 (7.6–8.8)	8.0 (7.5–8.8)	0.196
Lactate (mmol/L)	2.0 (1.3–2.9)	3.0 (1.7–3.9)	< 0.001
Albumin (g/dL)	3.0 (2.5–3.4)	2.0 (2.3–3.1)	< 0.001
Creatinine (mg/dL)	1.0 (0.9–2.0)	1.0 (0.9–2.2)	0.238
Total bilirubin (mg/dL)	1.0 (0.4–1.2)	3.0 (0.4–2.6)	< 0.001
Scoring system			
APACHEII (IQR)	13 (10–16)	15 (12–18)	< 0.001
SOFA (IQR)	2 (1–4)	3 (2–5)	< 0.001
Clinical outcomes			
LOS in hospital (days)	16 (6–20)	8 (3–13)	< 0.001

*SBP* systolic blood pressure, *DBP* diastolic blood pressure, *HR* heart rate, *RR* respiratory rate, *CAD* coronary artery disease, *WBC* white blood cells, *PLT* platelet, *RDW* red cell volume distribution width, *PT* prothrombin time, *PTT* partial thrombin time, *ALT* alanine aminotransferase, *AST* aspartate aminotransferase, *SOFA* sequential organ failure assessment, *APACHE *acute physiology and chronic health evaluation, *LOS* length of stay, *IQR* interquartile ranges, *MCV* mean corpuscular volume, *NLR* is defined as the ratio of neutrophils to lymphocytes

11 variables were enrolled in multivariable logistic regression analysis and 9 variables were identified as independent factors associated with 30-day mortality (Table 4): age(odds ratio (OR) 1.030,95% CI 1.020–1.039), AST(OR 1.000, 95% CI 1.000–1.001), urea nitrogen(OR 1.008,95% CI 1.004–1.013), RDW(OR 1.161, 95% CI 1.098–1.227), lactate(OR = 1.189, 95% CI 1.115–1.268), albumin(OR 0.581, 95% CI 0.447–0.708), total bilirubin(OR 1.059, 95% CI 1.029–1.091), PT(OR 1.031, 95% CI 1. 010–1.052) and PLT(OR 0.999, 95% CI 0.998–1.000).

**Table 4:** Multivariate logistic regression analysis of variables associated with 30-day mortality

Variables	B	S.E	Wald	*P* value	OR	95% CI	
						Lower	Upper
Age	0.029	0.005	38.973	< 0.001	1.030	1.020	1.039
AST	0.001	0.001	8.445	0.004	1.000	1.000	1.001
Urea nitrogen	0.008	0.002	14.690	< 0.001	1.008	1.004	1.013
RDW	0.149	0.028	27.483	< 0.001	1.161	1.098	1.227
Lactate	0.173	0.033	27.711	< 0.001	1.189	1.115	1.268
Albumin	−0.542	0.101	29.089	< 0.001	0.581	0.477	0.708
Total bilirubin	0.058	0.015	14.879	< 0.001	1.059	1.029	1.091
PT	0.030	0.010	8.775	0.003	1.031	1.010	1.052
PLT	−0.001	0.001	5.356	0.021	0.999	0.998	1.000

*AST* aspartate aminotransferase, *RDW* red cell volume distribution width, *RR* respiratory rate, *PLT* platelet, *PT* prothrombin time

### ANN model development

**Fig. 2:**
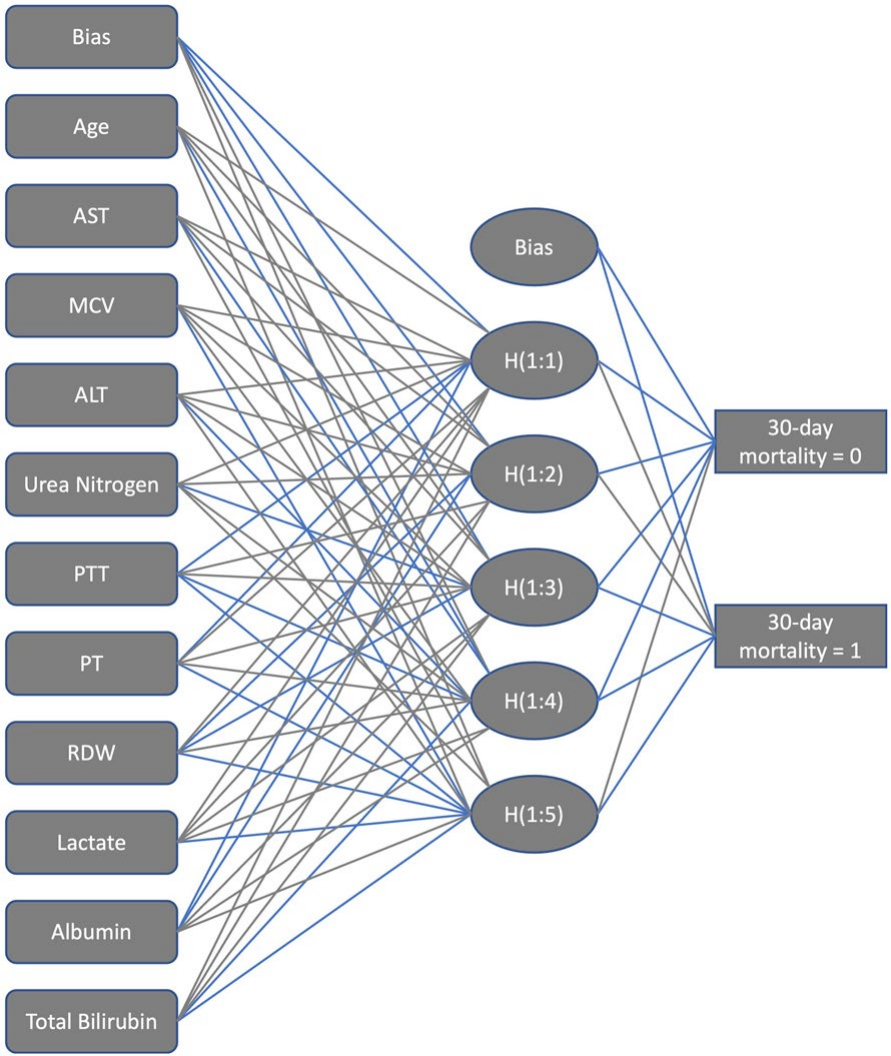
The main structures of artificial neural networks. *RDW* red cell volume distribution width, *PT* prothrombin time, *PTT* partial thrombin time, *ALT* alanine aminotransferase, *AST* aspartate aminotransferase, *MCV* mean corpusular volume

**Fig. 3:**
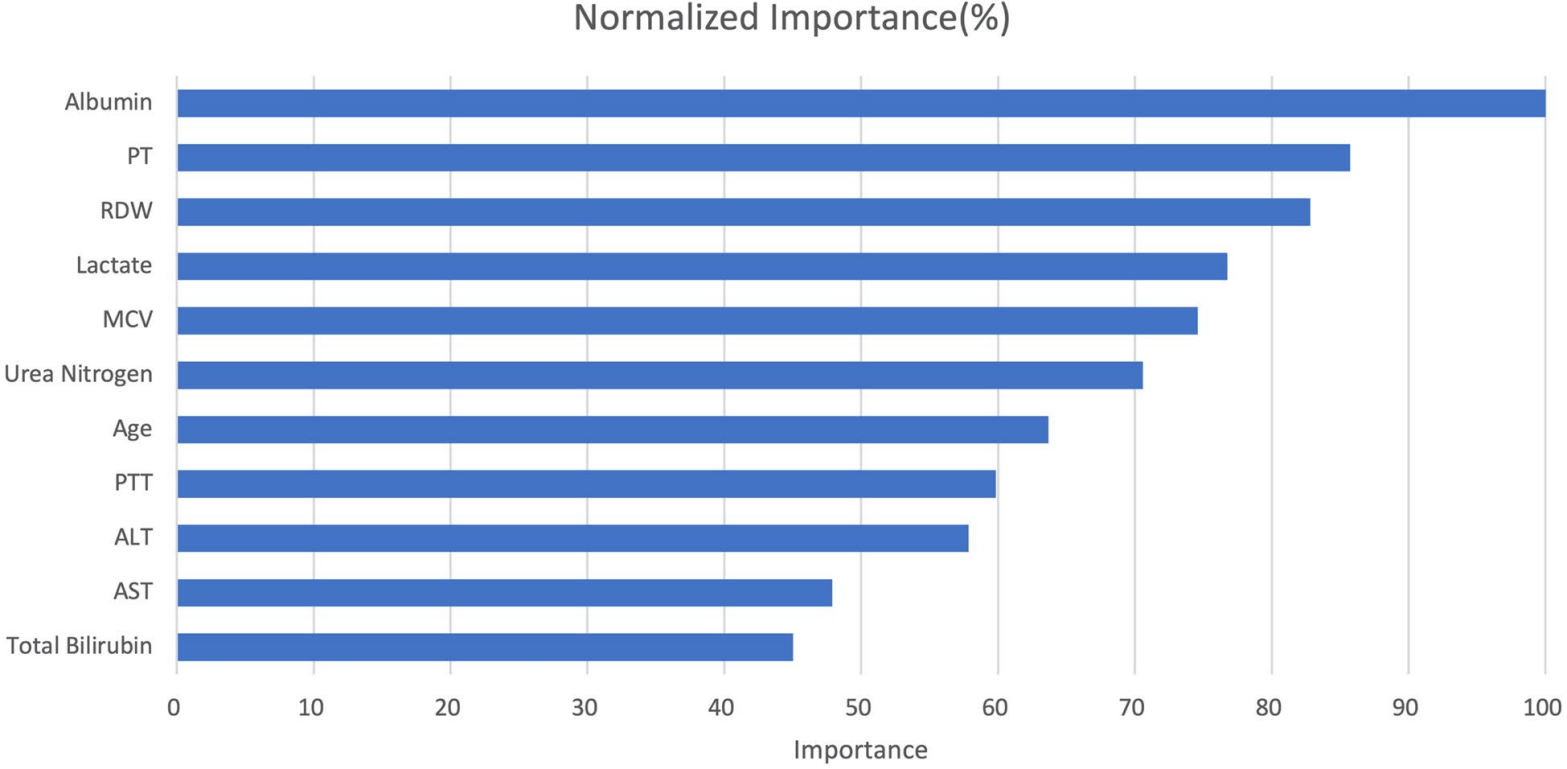
The normalized importance of 11 variables for predicting 30-day mortality by artificial neural networks. *RDW* red cell volume distribution width, *PT* prothrombin time, *PTT* partial thrombin time, *ALT* alanine aminotransferase, *AST* aspartate aminotransferase, *MCV* mean corpusular volume

The main structures of artificial neural networks were illuminated in Fig. 2. 11 variables including age, AST, MCV, ALT, urea nitrogen, PTT, PT, RDW, lactate, albumin and total bilirubin which showed significant differences between two groups were selected for the input layer. The output layer was 30-day hospital mortality. In Fig. 3, normalized importance of all 11 variables were demonstrated. The top four significant variables were albumin (100.00%), PT (85.73%), RDW (82.81%), and lactate (76.75%).

Predictive performance of different models in Training set and Validation set In Table 5, predictive performance of ANN, logistic regression, APAHCEII and SOFA scores for training set and validation set were demonstrated. In training set, the accuracies of the four models were 0.866, 0.711, 0.615, and 0.574, respectively (*P* < 0.001). The sensitivities were 0.850, 0.662, 0.569, and 0.619, respectively (*P* < 0.001). The specificities were 0.410, 0.337, 0.367 and 0.413, respectively (*P* = 0.029). The area under the ROC curve (AUC) of ANN, LR, APACHEII and SOFA scores were 0.873, 0.720, 0.629 and 0.619, respectively (*P* < 0.001). In validation set, the accuracies of the four models were 0.735, 0.722, 0.401, and 0.609, respectively (*P* = 0.272). The sensitivities were 0.624, 0.604, 0.333, and 0.416, respectively (*P* = 0.197). The specificities were 0.772, 0.744, 0.841, and 0.788, respectively (*P* = 0.095). The AUCs of ANN, LR, APACHEII, and SOFA scores were 0.811, 0.752, 0.607, and 0.628, respectively (*P* = 0.002).

**Table 5:** Predictive performances of different models in training set and validation set

	Accuracy (95% CI)	Sensitivity (95% CI)	Specificity (95% CI)	AUC (95% CI)
Training set				
ANN	0.866 (0.838–0.894)	0.850 (0.821–0.879)	0.410 (0.370–0.450)	0.873 (0.846–0.900)
Logistic regression	0.711 (0.674–0.748)	0.662 (0.624–0.700)	0.337 (0.299–0.375)	0.720 (0.684–0.756)
APACHEII	0.615 (0.576–0.654)	0.569 (0.529–0.609)	0.367 (0.328–0.406)	0.629 (0.607–0.651)
SOFA	0.574 (0.534–0.614)	0.619 (0.580–0.658)	0.413 (0.373–0.453)	0.619 (0.596–0.641)
* P* value	< 0.001	< 0.001	0.029	< 0.001
Validation set				
ANN	0.735 (0.714–0.756)	0.624 (0.601–0.647)	0.772 (0.752–0.792)	0.811 (0.792–0.830)
Logistic regression	0.722 (0.701–0.743)	0.604 (0.581–0.627)	0.744 (0.723–0.765)	0.752 (0.731–0.773)
APACHEII	0.401 (0.378–0.424)	0.333 (0.311–0.355)	0.841 (0.824–0.858)	0.607 (0.584–0.630)
SOFA	0.609 (0.586–0.632)	0.416 (0.392–0.440)	0.788 (0.769–0.807)	0.628 (0.605–0.651)
* P* value	0.272	0.197	0.095	0.002

*ANN* artificial neural networks, *SOFA* sequential organ failure assessment, *APACHE* acute physiology and chronic health evaluation, *AUC* area under the ROC curve, *CI* confidential interval

**Fig. 4:**
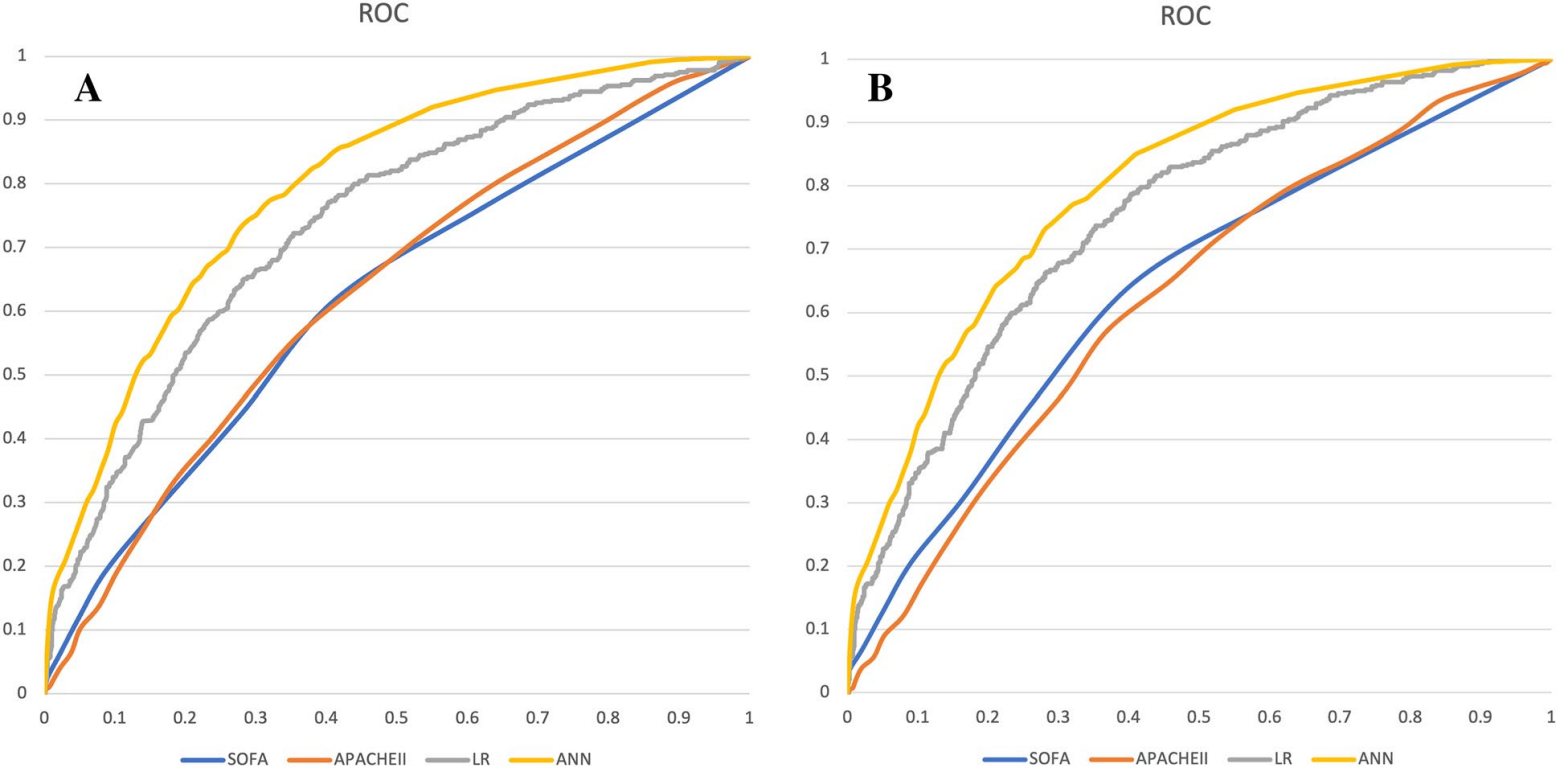
The receiver operating characteristic curves of ANN, LR, SOFA, APACHEII in predicting 30-day mortality in sepsis. **4A**: Training set; **4B**: Validation set. *ANN* artificial neural networks, *SOFA* sequential organ failure assessment, *APACHE* acute physiology and chronic health evaluation, *LR* logistic regression

Comparison of the predictive performances in different models Figure 4 showed the ROCs of ANN, LR, APACHEII, and SOFA scores for training set (A) and validation set (B), which showed that the ANN model had the highest ROCs in both training set and validation set. In Table 6, AUCs of ANN, LR, APACHEII and SOFA scores between training set and validation set were compared. ANN model showed the significant difference (*P* < 0.001), while no significant difference was found in logistic regression (*P* = 0.067), APACHEII score (*P* = 0.174) and SOFA score (*P* = 0.350).

**Table 6:** Comparison of predictive performance between training set and validation set

	AUC (95% CI, Training set)	AUC (95% CI, Validation set)	*P* value
ANN	0.873 (0.846–0.900)	0.811 (0.792–0.830)	< 0.001
Logistic regression	0.720 (0.684–0.756)	0.752 (0.731–0.773)	0.067
APACHEII	0.629 (0.607–0.651)	0.607 (0.584–0.630)	0.174
SOFA	0.619 (0.596–0.641)	0.628 (0.605–0.651)	0.350

*ANN* artificial neural networks, *SOFA* sequential organ failure assessment, *APACHE* acute physiology, and chronic health evaluation, *AUC* area under the ROC curve, *CI* confidential interval

## Discussion

In our study, an ANN model for predicting 30-day mortality in sepsis was performed. To our best knowledge, it was the first study for investigating the performance of ANN model in predicting short-term outcomes in sepsis based on MIMIC-III database.

Compared to LR model, ANN was good at dealing with nonlinear correlation in different analyses and also had a superiority in analysis of variables with sophisticated correlations [17]. One Korean study clarified that a total of 1260 bacteremia episodes were identified in 13,402 patients and ANN model had a better performance in early detection of bacteremia, with an AUC of 0.729 and a sensitivity of 0.810 [18]. Another study concluded that when ANN model was applied to the prediction of individual episodes of apnea and hypopnea in people with obstructive sleep apnea syndrome, it had both good specificity and sensitivity [19]. Our study showed that ANN model with an AUC of 0.811 was significantly superior to compared to LR, SOFA score and APACHEII score.

Four most important variables including albumin, PT, RDW, and lactate were identified in our ANN model. Accumulating evidence demonstrated those four variables were associated with clinical outcomes in sepsis [20–22].

Albumin, as the main protein which can balance capillary membrane permeability and plasma osmotic pressure, was identified to be associated with occurrence and clinical outcomes in sepsis [23]. One study clarified that low serum albumin levels (< 29.2 g/L) was an independent risk factor for 28-day mortality in sepsis [24]. Furthermore, the daily changes of albumin were significantly linked with mortality during the ICU stay in sepsis patients [25]. Another retrospective study concluded that in sepsis, the probability of survival decreased by 63.4% when serum albumin was ≤ 2.45 g/dl on admission, and by 76.4% when the lowest serum albumin during hospitalization was ≤ 1.45 g/dl [26].

Previous research illuminated that coagulation function on ICU admission was associated with mortality in sepsis [21]. In septic shock, survival curve analysis demonstrated a higher of PT/INR (> 0.16) had significantly higher risk in 28-day mortality compared with a lower level (< 0.16) [27]. One recent COVID-19 study found that non-survivors with sepsis had higher level of PT and APTT [28]. In sepsis, due to infection and activated innate immune system, coagulation will be activated, leading to sepsis associated coagulopathy with over-consumption of coagulation factors [29].

RDW, as a parameter for evaluating in the size of circulating red blood cells, was to be identified as a predictive indicator in different disorders [30–33]. A sepsis study with a total of 566 patients with overall mortality of 29% demonstrated that higher RDW was independently associated with 28-day mortality [34]. Another study investigated the association between RDW and in-hospital mortality in sepsis and found that RDW had good predictive performance with the AUC of 0.867 [35]. In a study on sepsis-induced acute respiratory distress syndrome, cox regression model showed that RDW was also an independent prognostic marker [36].

Lactate was reported as a predictor for the risk of death in all patients with or without sepsis [37]. Hyperlactatemia was more frequent in septic shock and was associated with a lower survival rate [38]. A prospectively research with a cohort of 1233 adults in UK showed that a lactate ≥ 2 mmol/L was associated with an increase in mortality and identified patients with suspicion of sepsis who had the highest risk of in-hospital mortality [39]. Lactate showed the similar prognostic accuracy for mortality in adults with sepsis compared to that of SOFA [4]. The current research proved that in polymicrobial sepsis, lactate could promote macrophage high mobility group box-1(HMGB1) lactylation/acetylation and release exosome, leading to disrupted endothelium integrity and increased vascular permeability [40].

In our study, we performed a predictive model for 30-day mortality in sepsis using ANN. Our predictive model can be beneficial for the early detection of patients with higher risk of poor prognosis. When those patients with higher risk of mortality are identified, physicians can do some intervention and timely managements in order to improve the clinical outcomes. Although the predictive model couldn’t help guide ICU management, it may be more relevant to target short-term outcomes including respiratory failure or vasopressor initiation within 48 h which could impact disposition decisions.

Some limitations should be stated in our study. First, the MIMIC-III public database included data before 2012, while the new definition of Sepsis-3.0 was published in 2016. Differences in the definition of sepsis in different phrases should be considered when applying our ANN model. Second, due to a high percentage of missing values in MIMIC-III, not all the variables which may affect the clinical outcomes in sepsis were included and analyzed. Some variables including the percentage of patients that received antibiotics, and the timing of such were not analyzed, which may confound the outcome of 30-day mortality. Third, the ANN model was applied to perform this study. Whether other prediction models of machine learning have better predictive performance than the ANN model should be further investigated. Fourth, our study constructed a predictive ANN model for 30-day mortality in sepsis. The primary outcome was 30-day mortality and patients with out-of-hospital mortality within 30 days might be missed. Fifth, we only investigated the 30-day mortality as the main outcome in the study. Other outcomes including complications and long-term prognosis were not investigated. In the future, further studies including more samples and longer follow-up should be conducted to help explore how to improve the clinical outcomes in sepsis.

## Conclusion

In our study, an ANN model for predicting 30-day mortality in sepsis was performed. The predictive model can be beneficial for the early detection of patients with higher risk of poor prognosis.

## Data Availability

The datasets used and/or analyzed during the present study were availed by the corresponding author on reasonable request.

## Abbreviations

SOFA: Sequential organ failure assessment
APACHE: Acute physiology and chronic health evaluation
LOS: Length of stay
ICU: Intensive care unit
CAD: Coronary artery disease
SBP: Systolic blood pressure
DBP: Diastolic blood pressure
HR: Heart rate
RR: respiratory rate
WBC: White blood cells
PLT: Platelet
RDW: Red cell volume distribution width
PT: Prothrombin time
PTT: Partial thrombin time
ALT: Alanine aminotransferase
INR: International normalized ratio
AST: Aspartate aminotransferase
MCV: Mean corpuscular volume
LOS: Length of stay
ICU: Intensive care unit
IQR: Interquartile ranges
CI: Confidential interval
OR: Odds ratio
AUC: Area under the curve
ROC: Receiver-operator characteristic
HMGB1: High mobility group box-1
